# The enzymatic hydrolysates from deer sinew promote MC3T3-E1 cell proliferation and extracellular matrix synthesis by regulating multiple functional genes

**DOI:** 10.1186/s12906-021-03240-2

**Published:** 2021-02-10

**Authors:** Zhenwei Zhou, Daqing Zhao, Pengcheng Zhang, Mei Zhang, Xiangyang Leng, Baojin Yao

**Affiliations:** 1 Jilin Ginseng Academy, Changchun University of Chinese Medicine, Changchun, 130117 China; 2 The Affiliated Hospital of Changchun University of Chinese Medicine, Changchun, 130117 China; 3 Innovation Practice Center, Changchun University of Chinese Medicine, Changchun, 130117 China

**Keywords:** Deer sinew, Enzymatic hydrolysates, MC3T3-E1 cells, RNA sequencing, Differentially expressed genes, Molecular mechanism

## Abstract

**Background:**

Deer Sinew serves as a medicinal food, and has been used for treating skeletal diseases, especially bone diseases in a long history. Thus, it could become an alternative option for the prevention and therapeutic remedy of bone-related diseases. In our previous study, we established an optimal extraction process of the enzymatic hydrolysates from Chinese Sika deer sinews (DSEH), and we demonstrated that DSEH significantly promoted the proliferation of MC3T3-E1 cells (an osteoblast-like cell line) with a certain dose-effect relationship. However, the precise molecular mechanism of deer sinew in regulating bone strength is still largely unknown. The aim of this study was to explore the underlying molecular mechanism of DSEH on MC3T3-E1 cells proliferation and extracellular matrix synthesis.

**Methods:**

Preparation and quality control were performed as previously described. The effect of DSEH at different administrated concentrations on cell proliferation was measured using both CCK-8 and MTT assays, and the capacity of DSEH on extracellular matrix synthesis was detected by Alizarin red staining and quantification. The gene expression pattern change of MC3T3-E1 cells under the treatment of DSEH was investigated by RNA-seq analysis accompanied with validation methods.

**Results:**

We demonstrated that DSEH promoted MC3T3-E1 cell proliferation and extracellular matrix synthesis by regulating multiple functional genes. DSEH significantly increased the expression levels of genes that promoted cell proliferation such as Gstp1, Timp1, Serpine1, Cyr61, Crlf1, Thbs1, Ctgf, P4ha2, Sod3 and Nqo1. However, DSEH significantly decreased the expression levels of genes that inhibited cell proliferation such as Mt1, Cdc20, Gas1, Nrp2, Cmtm3, Dlk2, Sema3a, Rbm25 and Hspb6. Furthermore, DSEH mildly increased the expression levels of osteoblast gene markers.

**Conclusions:**

Our findings suggest that DSEH facilitate MC3T3-E1 cell proliferation and extracellular matrix synthesis to consolidate bone formation and stability, but prevent MC3T3-E1 cells from oxidative stress-induced damage, apoptosis and further differentiation. These findings deepened the current understanding of DSEH on regulating bone development, and provided theoretical support for the discovery of optional prevention and treatment for bone-related diseases.

## Background

Deer Sinew, a precious animal production, has been widely used in traditional Chinese remedies to support bone health for over 20 centuries. According to the theory of traditional Chinese medicine, Deer sinews are considered to be more effective than cattle sinews in nourishing kidney yang (shen yang) and strengthening bones and tendons [1]. Several studies have shown that deer sinew extract could prevent bone loss in osteoporosis model and reduce the injury risk of musculoskeletal system. By using an ovariectomized rat model of osteoporosis, the researchers demonstrated that deer sinew extract could significantly increase bone mineral density and serum hydroxyproline, and improve the histomorphometric parameters and mechanical indicators of bone [2]. Consistent with the above results, according to the investigation of a retinoic acid-induced rat model of osteoporosis, the researchers demonstrated that deer sinew extract could increase bone mineral density and bone weight [3]. In a clinical investigation of the effect of deer sinew supplementation on the exercise performance and risk training injury of a population of athletes, the researchers demonstrated that deer sinew extract could improve exercise performance and reduce the injury risk of musculoskeletal system [4]. Furthermore, several studies have also shown that deer sinew extract has potential anti-inflammatory effects [5–7]. Sinews, also known as tendons, are fibrous connective tissues that localize between bones and muscles [8]. The major components of sinews are collagens, especially type I collagen, which account for 65 to 80% of the dry mass [9]. Meanwhile, collagens including types I, III and V are the major extracellular matrices in bone, and type I collagen accounts for about 95% of the total collagen components present in bone and approximately 80% of the entire proteins of bone. Those collagens thus play pivotal roles in maintaining bone strength and further prevent bone fragility related diseases, such as osteogenesis imperfecta and osteoporosis [10]. However, the precise molecular mechanism of deer sinew in regulating bone strength is still largely unknown.

In recent years, high-throughput sequencing technology has led rapid increase in understanding the physiological and pathological mRNA dynamics, which further influence the protein expression that involved in the growth, development and related functions of cells as well as organ systems [11]. It is particularly noteworthy that RNA sequencing (RNA-seq) has also been widely used in skeletal biology research to solve tough problems regarding the development and disease control of skeletal tissues, including bone, cartilage, tendon and ligament [12]. During bone formation and repair, osteoblasts serve as bone-forming cells that are required for the synthesis of bone matrix, including collagen proteins (mainly type I collagen), noncollagen proteins (osteocalcin, osteonectin, bone sialoprotein II and osteopontin), and proteoglycans (decorin and biglycan) [13, 14].

In our previous study, we established an optimal extraction process of the enzymatic hydrolysates from Chinese Sika deer sinews (DSEH). The yield of DSEH was 58.56%, and the protein concentration was 48.4%. Cell proliferation assay showed that DSEH significantly promoted the proliferation of MC3T3-E1 cells (an osteoblast-like cell line) with a certain dose-effect relationship, and the proliferative effect was most significant at an administration concentration of 8 mg/ml [15]. Therefore, in the present study, we performed RNA-seq analysis accompanied with validation methods to explore the underlying molecular mechanism of DSEH on MC3T3-E1 cells via deeply dissecting the gene expression patterns under DSEH treatment. We demonstrated that DSEH promoted MC3T3-E1 cell proliferation and extracellular matrix synthesis by regulating multiple functional genes. DSEH significantly increased the expression levels of genes that promoted cell proliferation such as Gstp1, Timp1, Serpine1, Cyr61, Crlf1, Thbs1, Ctgf, P4ha2, Sod3 and Nqo1. However, DSEH significantly decreased the expression levels of genes that inhibited cell proliferation such as Mt1, Cdc20, Gas1, Nrp2, Cmtm3, Dlk2, Sema3a, Rbm25 and Hspb6. Furthermore, DSEH mildly increased the expression levels of osteoblast gene markers. Our findings deepened the current understanding of DSEH on regulating bone development, and provided theoretical support for the discovery of optional prevention and treatment for bone-related diseases.

## Methods

### DSEH treatment and cell proliferation assay

Deer sinews were purchased from the Shuangyang deer farm in Changchun, China. The preparation of the enzymatic hydrolysates from Chinese Sika deer sinews (DSEH) was carried out as previously described [15]. All procedures were submitted and approved by the ethics committee of Changchun University of Chinese Medicine in accordance with the guidelines of the ethical protocol (No. ccucm-2017-0015). Primary osteoblasts from individual calvaria of newborn C57BL/6 mice (*n* = 6) were isolated according to the previous protocol [16]. Primary osteoblasts and MC3T3-E1 cells (ATCC, USA) were inoculated into a 96-well cell culture plate (Thermo, USA) at a cell density of 5 × 10^3^ cells/well, respectively, and incubated in a humidified incubator (Thermo, USA) containing 5% CO_2_ at 37 °C for 12 h. The effects of DSEH at different administrated concentrations (0, 2 mg/ml, 4 mg/ml, 6 mg/ml, 8 mg/ml and 10 mg/ml) on cell proliferation were measured using a CCK-8 assay kit (Sigma, USA) and a MTT assay kit (Sigma, USA) according to the manufacturers’ protocols.

### Alizarin red staining and quantification

Primary osteoblasts and MC3T3-E1 cells were inoculated into a 24-well cell culture plate at a cell density of 2 × 10^4^ cells/well, and incubated in a humidified incubator (Thermo, USA) containing 5% CO_2_ at 37 °C for 24 h. The cells were either treated by DSEH at an optimum concentration based on the result of the cell proliferation assay or treated with plain culture medium (Thermo, USA), and incubated for 24 h. All culture mediums were removed, and cells were washed with phosphate-buffered saline buffer (Thermo, USA). Cell fixation was performed at room temperature by incubating with 4% paraformaldehyde for 15 min. After washing with Millipore purified water for three times, the fixed cells were stained with 1% Alizarin red S staining solution (Solarbio, China) for 0.5 h at room temperature, and washed with purified water for three times. Images were taken with an optical microscope (Olympus, Japan) accompanied with a digital camera. Quantitative analysis was carried out by dissolving the stained cells with 10% acetic acid (Thermo, USA) at room temperature for 0.5 h with gentle shaking, followed by vortexing, heating, centrifuging and neutralizing by ammonium hydroxide, and quantified using a plate reader (Life science, USA) at an optical density (OD) of 405 nm [17].

### RNA purification and quality control

MC3T3-E1 cells were inoculated into a 6-well cell culture plate at a cell density of 1 × 10^6^ cells/well, and incubated in a humidified incubator (Thermo, USA) containing 5% CO_2_ at 37 °C for 12 h. The cells were either treated with DSEH at an optimum concentration based on the result of the cell proliferation assay or treated with plain culture medium (Thermo, USA). All culture mediums were removed, and cells were gently rinsed with precooled phosphate-buffered saline buffer (Thermo, USA). Total RNA from experiments in triplicate was isolated with TRIzol reagent (Invitrogen, USA) according to the manufacturer’s instructions. The total RNA was qualified and quantified based on the RNA integrity number (RIN) measured by an Agilent Bioanalyzer 2100 (Agilent Technologies, USA) following the instrument’s protocols.

### Library construction and sequencing

The RNA-seq libraries were generated using a TruSeq Stranded mRNA kit (Illumina, USA) in accordance with the company’s recommendations. Briefly, the messenger RNA containing poly (A) was purified from the total RNA using a magnetic poly (T) beads. Messenger RNA was broke into short fragments with a fragmentation buffer. Double stranded cDNA was synthesized by random hexamer primers, following by purification, repair and sequencing adapter ligation, and amplified by polymerase chain reaction to generate the libraries. The libraries were sequenced by performing high-throughput sequencing via the Illumina HiSeq 2500 platform with a paired-end read length of 150 bp (Illumina, USA).

### Sequencing data analysis

After illumine sequencing, the image data was transformed via base calling into raw data in FASTQ format. The raw data were processed using Perl scripts to generate clean reads by removing the low-quality reads and adapter sequences, following by Q30 evaluation and GC content calculation. The data sets were submitted into the NCBI Sequence Read Archive (SRA) database with an accession number PRJNA612675. Subsequently, the clean reads were aligned with the mouse (*Mus musculus*) reference genome using the HISAT program [18]. The gene expression levels of each transcript were estimated according to the FPKM algorithm [19]. The BLAST program was used to perform annotation against the Non-Redundant (NR) and Swiss-Prot protein databases [20]. The differential expression between the DSEH treated group and untreated group were analyzed by the DEGseq program [21]. Differentially expressed genes (DEGs) were identified using the following criteria: log2 fold change ≥1 or ≤ − 1 and with a *p* value ≤0.001.

### Enrichment analysis

The enrichment analysis of Gene ontology (GO) and Kyoto Encyclopedia of Genes and Genomes (KEGG) was conducted by R function phyper to determine the biological functions and signaling pathways of those identified DEGs. The significance of enriched functional clusters and signaling pathways were determined based on the adjusted *p* value (Q value) of less than 0.05, which was calculated according to the Hypergeometric test and Bonferroni correction [22].

### Quantitative real-time PCR (qRT-PCR) verification

The RNA-seq results were further verified through examining the expression levels of DEGs by qRT-PCR assay. Briefly, total RNA was extracted using the TRIzol reagent (Invitrogen, USA) in accordance with the manufacturer’s protocols. Reverse-transcribed cDNA was synthesized using the iScript cDNA Synthesis kit (Bio-Rad, USA), following by qRT-PCR detection under standard amplification condition using a generic SYBR_ Green Supermix kit (Bio-Rad, USA) on a CFX Connect Real-Time PCR Detection System (Bio-Rad, USA). The gene of mouse glyceraldehyde 3-phosphate dehydrogenase (Gapdh) was selected as the internal reference gene. The normalized expression of validated genes was calculated based on the 2^−ΔΔCT^ method [23].

### Effects of DSEH on osteoporotic model caused by hydrogen peroxide (H_2_O_2_)-induced oxidative injury

Effects of DSEH on MC3T3-E1 cells under hydrogen peroxide (H_2_O_2_)-induced oxidative injury were evaluated to further validate the regulatory function of DSEH on osteoblasts [24]. Briefly, MC3T3-E1 cells were either treated with 500 μM H_2_O_2_ for 6 h (H_2_O_2_ group), or treated with 500 μM H_2_O_2_ for 6 h followed by the addition of 8 mg/ml DSEH (H_2_O_2_ + DSEH group), untreated MC3T3-E1 cells serve as a negative control (Blank group). Cells were double stained with propidium iodide (PI) and Annexin V-FITC using a BD Apoptosis Detection Kit (Thermo, USA), and analyzed by a FlowSight® Imaging Flow Cytometer (Merck Millipore, USA). Analysis of cell apoptosis was performed using the IDEAS Application V6.1 software (Amnis, USA).

## Results

### DSEH promoted MC3T3-E1 cell and primary osteoblast proliferation in a dose-dependent manner

**Fig. 1:**
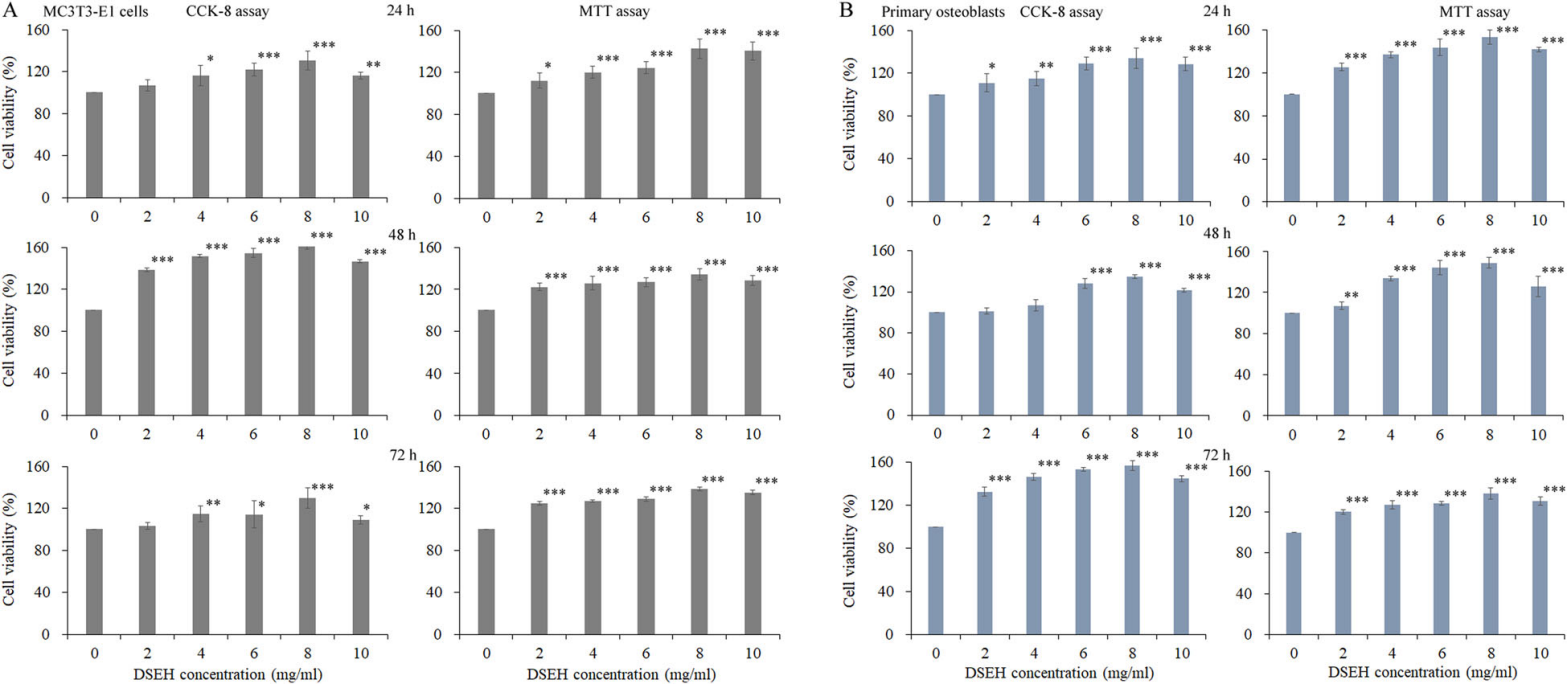
Effects of DSEH on the cell proliferation of MC3T3-E1 cells and primary osteoblasts. **a** MC3T3-E1 cells. **b** Primary osteoblasts. Cell viabilities were detected by CCK-8 and MTT assays under the treatment of DSEH at progressively increasing concentrations of 0 mg/ml, 2 mg/ml, 4 mg/ml, 6 mg/ml, 8 mg/ml and 10 mg/ml) at different time points (24 h, 48 h, 72 h). Cell viabilities of the DSEH treatment groups under different concentrations were estimated by normalizing to that of the untreated group (0 mg/ml). Data were presented as the mean with standard deviation for technical triplicate in an experiment representative of several independent ones (*n* = 4). *p* < 0.05, *p* < 0.01 and *p* < 0.001 represented the differences in cell viabilities under DSEH treatment in a student t-test

We first investigated the effects of DSEH on the cell proliferation of MC3T3-E1 cells and primary osteoblasts. The results of CCK-8 assay showed that DSEH promoted the proliferation of MC3T3-E1 cells and primary osteoblasts in a dose-dependent manner, as shown in Fig. 1. Compared with the untreated group (0 mg/ml), the viabilities of both cells were significantly increased under the DSEH treatment at progressively increasing concentrations. Meanwhile, the results of MTT assay also showed similar results as those of CCK-8 assy. Since DSEH treatments at the concentration of 8 mg/ml had the highest cell viability, thus, in the following experiments, the concentration of DSEH treatment was selected as 8 mg/ml.

### DSEH enhanced MC3T3-E1 cell extracellular matrix synthesis in a time-dependent manner

**Fig. 2:**
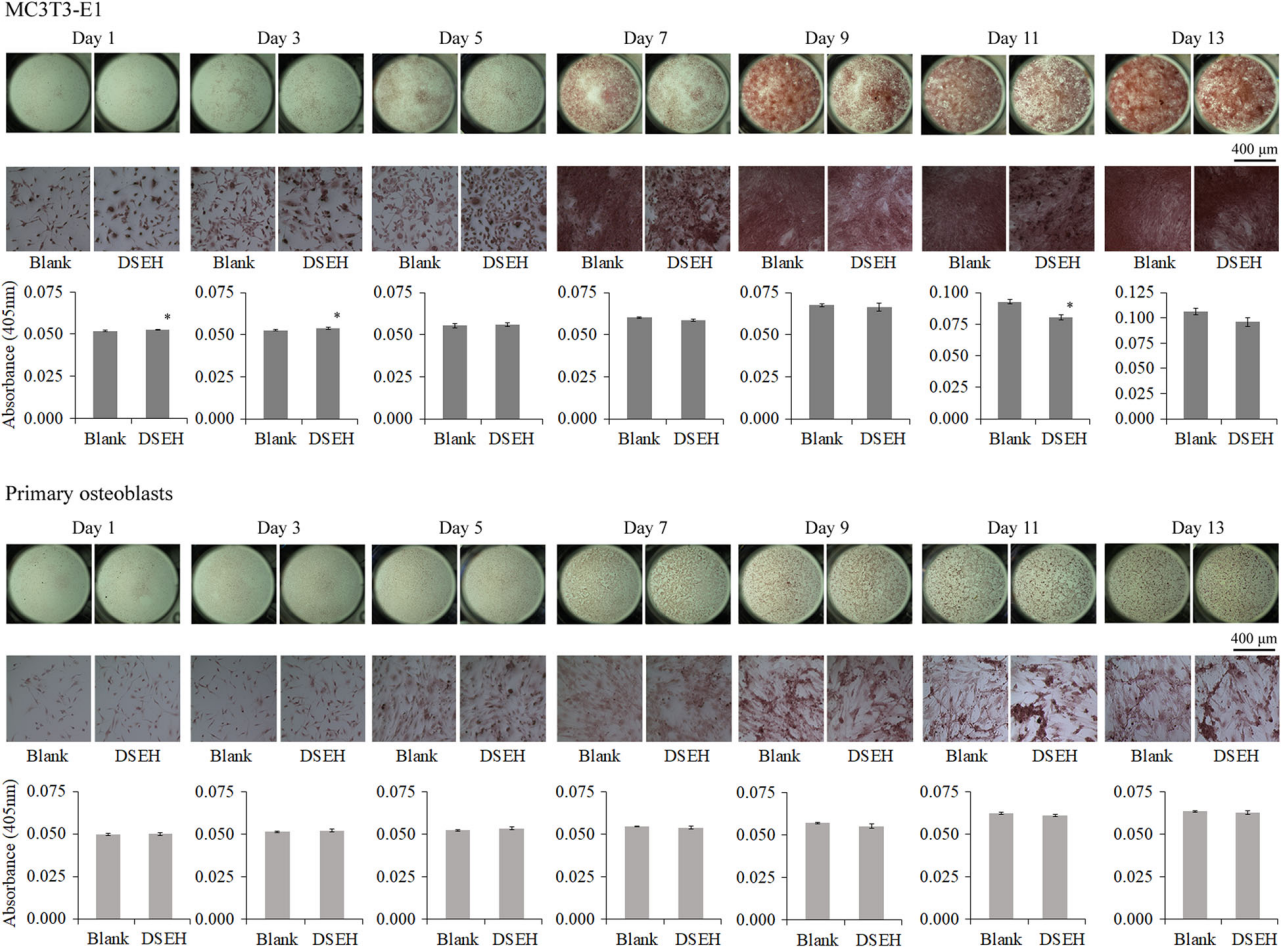
Effects of DSEH on extracellular matrix synthesis of primary osteoblasts and MC3T3-E1 cells. **a** Alizarin red staining and quantification of MC3T3-E1 cells at different time points (Day 1, 3, 5, 7, 11, 13) with or without DSEH treatment. **b** Alizarin red staining and quantification of primary osteoblasts at different time points (Day 1, 3, 5, 7, 11, 13) with or without DSEH treatment. The deposition of extracellular matrix of cells was visualized by Alizarin red S staining. The amount of Alizarin red staining was quantified by detecting absorbance of 405 nm

In order to investigate the effects of DSEH on osteoblast extracellular matrix synthesis, we performed Alizarin red staining and quantification at different time points as follows, 1 d, 3 d, 5 d, 7 d, 11 d and 13 d. As shown in Fig. 2, DSEH significantly enhanced extracellular matrix synthesis of MC3T3-E1 cells at day 1 and day 3 with a similar pattern, but inhibit extracellular matrix synthesis of MC3T3-E1 cells from day 7 to day 13. However, DSEH showed no significant effects on extracellular matrix synthesis of primary osteoblasts.

### Gene expression analysis of MC3T3-E1 cells with or without DSEH treatment was performed by RNA sequencing, genome mapping, and transcript annotation

To explore the molecular mechanism underlying the effects of DSEH on MC3T3-E1 cells, we performed RNA-seq analysis on MC3T3-E1 cells with or without DSEH treatment. As shown in Table 1, after Illumina sequencing and data processing, 62,258,284 and 42,159,210 clean reads were obtained from MC3T3-E1 cells treated with plain medium (Blank group) and those treated with DSEH (DSEH group), respectively. The quality assessment showed that the Q30 percentages were more than 92%, and the GC content percentages were approximately 52%. For the Blank and DSEH-treated samples, 59,578,848 and 40,340,992 clean reads were mapped to the mouse genome, respectively. In total, 13,962 out of 14,295 (Blank group), and 14,057 out of 14,373 (DSEH group) transcripts were annotated by searching against the Non-Redundant (NR) NCBI protein database and Swiss-Prot protein database, respectively.

**Table 1:** Statistic overview of sequencing and mapping results

Statistics	Blank group	DSEH group
Clean reads	62,258,284	42,159,210
Q30 percentage	92.44	92.56
GC percentage	51.77	52.05
Total mapped reads	59,578,848	40,340,992
Total transcripts	14,295	14,373
Known transcripts	13,962	14,057

### A series of DEGs were identified in MC3T3-E1 cells with or without DSEH treatment

Differential expression analysis was carried out to discover DEGs significantly changed under DSEH treatment. As shown in Table 2, in total, 841 genes with significant differences in the expression levels between DSEH group and Blank group were identified. Among those identified DEGs, 494 DEGs were significantly up-regulated, and 347 genes were significantly down-regulated (DSEH group vs. Blank group). The top 20 up-regulated DEGs mainly consisted of glutathione S-transferase P1 (Gstp1), metalloproteinase inhibitor 1 (Timp1), plasminogen activator inhibitor 1 (Serpine1), protein CYR61 (Cyr61), H-2 class I histocompatibility antigen D-B alpha chain (H2d1) and connective tissue growth factor (Ctgf), etc., as shown in Table 3, whereas the top 20 down-regulated DEGs mainly consisted of metallothionein-1 (Mt1), non-histone chromosomal protein HMG-17 (Hmgn2), prolactin-2C2 (Prl2c2), cell division cycle protein 20 homolog (Cdc20), prolactin-2C3 (Prl2c3) and transmembrane glycoprotein NMB (Gpnmb), etc., as shown in Table 4.

**Table 2:** Statistic summary of DEGs (DSEH group vs. Blank group)

Statistics	Number
Differentially expressed mRNA	841
Upregulated mRNAs	494
Downregulated mRNAs	347

**Table 3:** List of the top 20 up-regulated DEGs (DSEH group vs. Blank group)

Gene name	Blank (FPKM)	DSEH (FPKM)	log_2_ fold change (DSEH /Blank)	*P* value
Glutathione S-transferase P1 (Gstp1)	367.61	855.07	1.22	2.30E-308
Metalloproteinase inhibitor 1 (Timp1)	286.17	601.03	1.07	2.54E-223
Plasminogen activator inhibitor 1 (Serpine1)	131.49	550.47	2.07	0
Protein CYR61 (Cyr61)	153.48	444.08	1.53	0
H-2 class I histocompatibility antigen, D-B alpha chain (H2d1)	173.13	359.46	1.05	2.90E-274
Connective tissue growth factor (Ctgf)	28.09	357.75	3.67	0
Radiation-inducible immediate-early gene IEX-1 (Ier3)	76.23	338.48	2.15	0
Cytokine receptor-like factor 1 (Crlf1)	75.46	270.42	1.84	0
Thrombospondin-1 (Thbs1)	94.94	248.54	1.39	0
Metalloproteinase inhibitor 3 (Timp3)	69.64	214.57	1.62	0
Thioredoxin reductase 1, cytoplasmic (Txnrd1)	98.89	206.75	1.06	0
Host cell factor C1 regulator 1 (Hcfc1r1)	80.00	206.17	1.37	8.01E-166
Cysteine and glycine-rich protein 2 (Csrp2)	91.15	201.85	1.15	1.22E-83
Serine protease HTRA1 (Htra1)	98.34	200.55	1.03	8.28E-177
Rho-related GTP-binding protein RhoB (Rhob)	78.30	199.78	1.35	3.74E-304
Thioredoxin-interacting protein (Txnip)	26.89	175.95	2.71	0
Heme transporter HRG1 (Slc48a1)	67.54	157.59	1.22	1.89E-230
Annexin A6 (Anxa6)	62.46	148.03	1.24	9.92E-235
Flavin reductase (NADPH)(Blvrb)	47.91	142.93	1.58	9.87E-95
Proline-rich protein 13 (Prr13)	47.48	135.82	1.52	1.47E-117

**Table 4:** List of the top 20 down-regulated DEGs (DSEH group vs. Blank group)

Gene name	Blank (FPKM)	DSEH (FPKM)	log_2_ fold change (DSEH /Blank)	*P* value
Metallothionein-1 (Mt1)	419.48	188.65	−1.15	6.40E-107
Non-histone chromosomal protein HMG-17 (Hmgn2)	310.59	145.14	−1.10	4.58E-176
Prolactin-2C2 (Prl2c2)	302.57	105.30	−1.52	9.84E-167
Cell division cycle protein 20 homolog (Cdc20)	118.90	57.15	−1.06	3.10E-94
Prolactin-2C3 (Prl2c3)	166.68	55.41	−1.59	1.69E-109
Transmembrane glycoprotein NMB (Gpnmb)	76.68	30.90	−1.31	1.28E-184
Neuropilin-2 (Nrp2)	45.13	22.36	−1.01	2.48E-107
Muscleblind-like protein 1 (Mbnl1)	58.40	19.94	−1.55	6.30E-240
Growth arrest-specific protein 1 (Gas1)	48.10	17.13	−1.49	5.60E-116
Protein PRRC2C (Prrc2c)	31.70	15.05	−1.07	1.73E-153
Monofunctional C1-tetrahydrofolate synthase, mitochondrial (Mthfd1l)	30.62	14.72	−1.06	6.77E-53
Sodium/potassium/calcium exchanger 3 (Slc24a3)	29.19	14.47	−1.01	7.42E-50
Protein DEK (Dek)	29.00	13.78	−1.07	5.39E-36
Semaphorin-3A (Sema3a)	27.29	13.47	−1.02	4.21E-85
High mobility group protein HMGI-C (Hmga2)	34.31	13.10	−1.39	1.50E-35
Mannose-P-dolichol utilization defect 1 protein (Mpdu1)	25.49	12.73	−1.00	1.24E-14
Lymphocyte-specific helicase (Hells)	25.43	11.80	−1.11	7.89E-39
CKLF-like MARVEL transmembrane domain-containing protein 3 (Cmtm3)	30.93	11.47	−1.43	5.24E-36
Homeobox protein engrailed-1 (En1)	32.58	11.37	−1.52	1.05E-65
Small subunit processome component 20 homolog (Utp20)	27.90	11.06	−1.33	1.98E-163

### DEGs were classified into a variety of functional categories by functional enrichment analysis

**Fig. 3:**
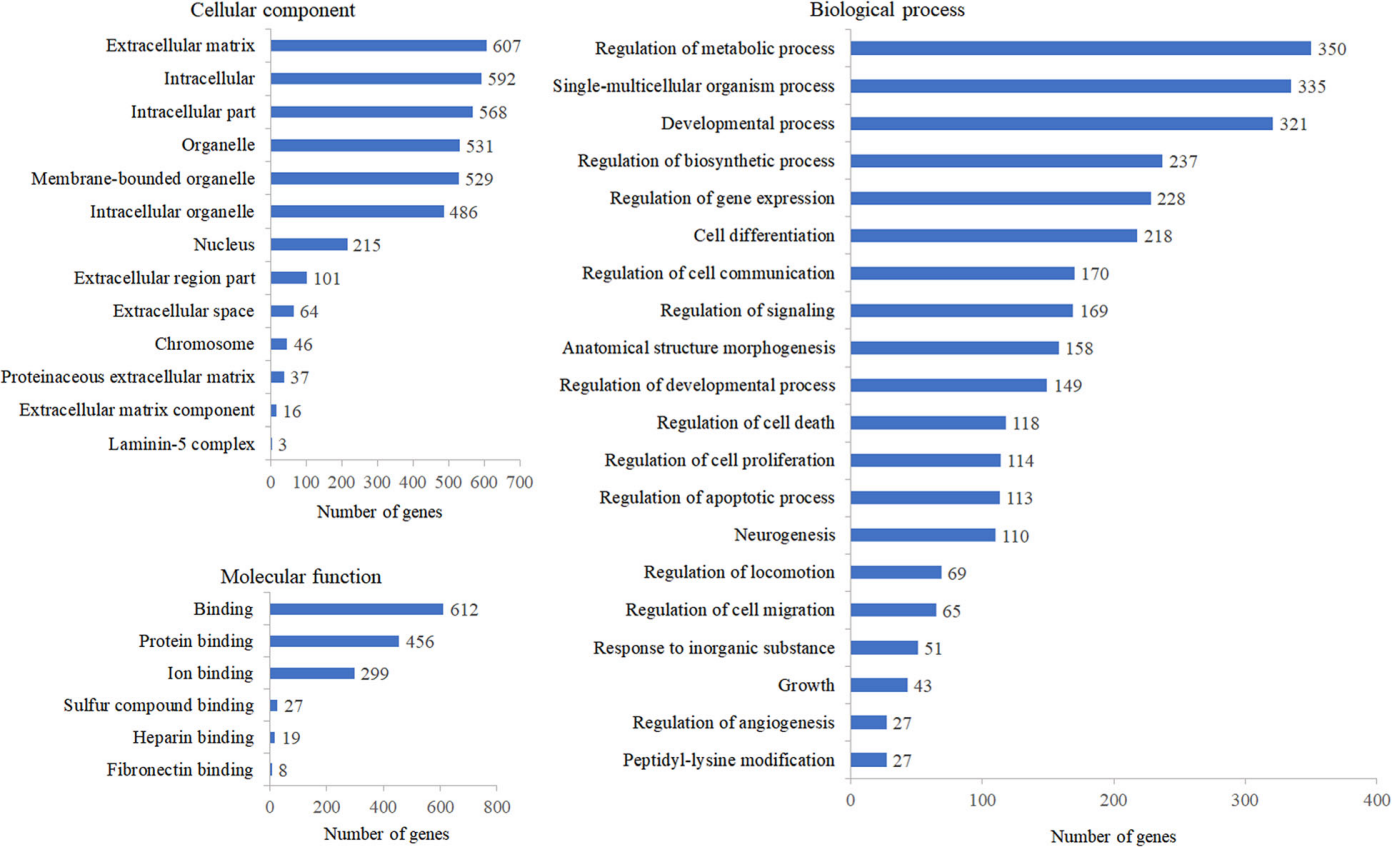
Histogram presentation of GO enrichment analysis of DEGs. The results were grouped into three main categories: cellular component, molecular function and biological process. The x-axis represents the number of DEGs corresponding to the GO terms, and the y-axis represents the name of the GO terms

GO enrichment analysis was performed to specify the distribution of these identified DEGs in cellular location, function and physiological processes. According to the functional enrichment analysis of DEGs, the identified DEGs were classified into the following GO categories: cellular component, molecular function and biological process, as shown in Fig. 3. Cellular component classification showed that most of the DEGs were located in the regions of extracellular matrix, intracellular part and organelle. Molecular function classification showed that the dominant functions of these DEGs were involved in binding activities, such as protein binding and ion binding. Biological process classification showed that these DEGs mainly participated in the biological processes including regulation of metabolic process, single-multicellular organism process and developmental process.

### DEGs were mapped into multiple signaling pathways by pathway enrichment analysis

**Fig. 4:**
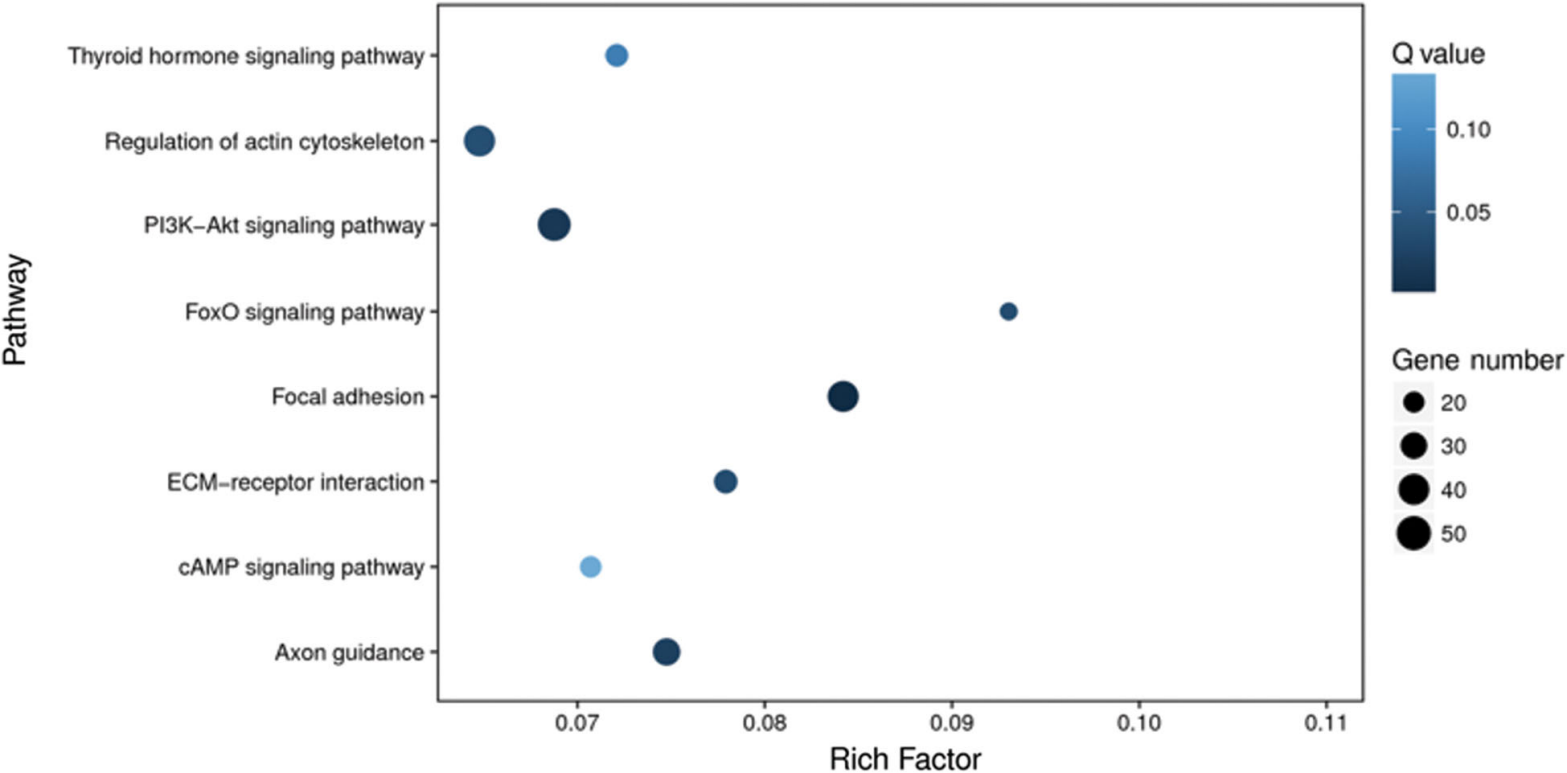
Scatterplot presentation of KEGG enrichment analysis of DEGs. The x-axis indicates the rich factor, representing the proportion of DEGs involved in the KEGG pathway in all the identified DEGs, and the y-axis indicates the enriched pathways. The size of the dots reflects the number of DEGs, and the color of the dots reflects the adjusted *p* value (Q value)

KEGG enrichment analysis was carried out to uncover the related signaling pathways by searching the identified DEGs against the KEGG database. As shown in Fig. 4, the DEGs were mainly mapped to the following nine signaling pathways, including thyroid hormone signaling pathway, regulation of actin cytoskeleton, PI3K-Akt signaling pathway, FoxO signaling pathway, focal adhesion, ECM-receptor interaction, cAMP signaling pathway and axon guidance.

### DSEH increased the expression levels of genes positively regulating the proliferation of MC3T3-E1 cells

Next, we screened the DEGs involved in positively regulating cell proliferation to further dissect the molecular mechanisms of DSEH on regulating MC3T3-E1 cells. Among the identified DEGs, there were 10 up-regulated DEGs, including glutathione S-transferase P1 (Gstp1), metalloproteinase inhibitor 1 (Timp1), metalloproteinase inhibitor 1 (Serpine1), protein CYR61 (Cyr61), cytokine receptor-like factor 1 (Crlf1), thrombospondin-1 (Thbs1), connective tissue growth factor (Ctgf), prolyl 4-hydroxylase subunit alpha-2 (P4ha2), extracellular superoxide dismutase (Sod3) and NAD(P) H dehydrogenase [quinone] 1 (Nqo1), which positively regulated the proliferation of osteoblasts, as shown in Table 5.

**Table 5:** List of DEGs positively regulating the proliferation of osteoblasts (DSEH vs. Blank)

Gene name	Blank (FPKM)	DSEH (FPKM)	log2 fold change (DSEH/Blank)	*P* value
Glutathione S-transferase P1 (Gstp1)	367.61	855.07	1.22	2.30E-308
Metalloproteinase inhibitor 1 (Timp1)	286.17	601.03	1.07	2.54E-223
Plasminogen activator inhibitor 1 (Serpine1)	131.49	550.47	2.06	0
Protein CYR61 (Cyr61)	153.48	444.08	1.53	0
Cytokine receptor-like factor 1 (Crlf1)	75.46	270.42	1.84	0
Thrombospondin-1 (Thbs1)	94.94	248.54	1.39	0
Connective tissue growth factor (Ctgf)	28.09	357.75	3.67	0
Prolyl 4-hydroxylase subunit alpha-2 (P4ha2)	59.23	121.92	1.04	9.15E-124
Extracellular superoxide dismutase (Sod3)	55.61	114.46	1.04	1.21E-103
NAD(P) H dehydrogenase [quinone] 1 (Nqo1)	34.57	107.55	1.64	1.21E-146

### DSEH decreased the expression levels of genes negatively regulating the proliferation of MC3T3-E1 cells

Then, we screened the DEGs involved in negatively regulating the proliferation of MC3T3-E1 cells. In consistent with the above results, among the identified DEGs, there were 9 down-regulated DEGs, including metallothionein-1 (Mt1), cell division cycle protein 20 homolog (Cdc20), growth arrest-specific protein 1 (Gas1), neuropilin-2 (Nrp2), CKLF-like MARVEL transmembrane domain-containing protein 3 (Cmtm3), semaphorin-3A (Sema3a), protein delta homolog 2 (Dlk2), RNA-binding protein 25 (Rbm25) and heat shock protein beta-6 (Hspb6), which negatively regulated the proliferation of osteoblasts, as shown in Table 6.

**Table 6:** List of DEGs negatively regulating the proliferation of osteoblasts (DSEH vs. Blank)

Gene name	Blank (FPKM)	DSEH (FPKM)	log2 fold change (DSEH/Blank)	*P* value
Metallothionein-1 (Mt1)	419.48	188.65	−1.15	6.40E-107
Cell division cycle protein 20 homolog (Cdc20)	118.9	57.15	−1.06	3.10E-94
Growth arrest-specific protein 1 (Gas1)	48.1	17.13	−1.49	5.60E-116
Neuropilin-2 (Nrp2)	45.13	22.36	−1.01	2.48E-107
CKLF-like MARVEL transmembrane domain-containing protein 3 (Cmtm3)	30.93	11.47	−1.43	5.24E-36
Protein delta homolog 2 (Dlk2)	29.22	9.04	−1.69	1.81E-39
Semaphorin-3A (Sema3a)	27.29	13.47	−1.02	4.21E-85
RNA-binding protein 25 (Rbm25)	20.1	7.94	−1.33	2.54E-57
Heat shock protein beta-6 (Hspb6)	19.42	8.86	−1.13	2.44E-14

### DSEH mildly increased the expression levels of osteoblast markers in MC3T3-E1 cells

Finally, we analyzed the expression levels of osteoblast markers under DSEH treatment in order to have a deeper recognition regarding the effects of DSEH on MC3T3-E1 cells. The results were consistent with our above findings that DSEH mildly increased the expression levels of multiple osteoblast marker genes including osteopontin (Spp1), collagen alpha-1(I) chain (Col1a1), fibronectin (Fn1), cyclic AMP-dependent transcription factor ATF-4 (Atf4), bone marrow stromal antigen 2 (Bst2) and bone morphogenetic protein 1 (Bmp1), etc., as shown in Table 7.

**Table 7:** Gene list of osteoblast markers

Gene name	Blank (FPKM)	DSEH (FPKM)	log2 fold change (DSEH/Blank)	*P* value
Osteopontin (Spp1)	1044.05	2006.86	0.94	0
Collagen alpha-1(I) chain (Col1a1)	1162.61	1354.5	0.22	3.44E-214
Fibronectin (Fn1)	520.41	1033.51	0.99	0
Cyclic AMP-dependent transcription factor ATF-4 (Atf4)	267.17	322.15	0.27	1.55E-21
Bone marrow stromal antigen 2 (Bst2)	21.42	28.88	0.43	2.93E-03
Bone morphogenetic protein 1 (Bmp1)	21.89	26.28	0.26	1.60E-05
Bone morphogenetic protein 4 (Bmp4)	14.42	17.55	0.28	7.66E-03
Transcription factor SOX-4 (Sox4)	13.14	15.95	0.28	3.01E-03
Transcription factor SOX-11 (Sox11)	5.89	8.68	0.56	4.50E-13
Homeobox protein MSX-1 (Msx1)	3.28	5.35	0.71	1.19E-03
Zinc finger protein 25 (Znf25)	3.29	4.83	0.55	2.87E-04
Osteocalcin-2 (Bglap2)	0.81	0.91	0.17	8.36E-01
Transcription factor Sp7 (Sp7)	0.13	0.81	2.64	1.84E-04
Osteocalcin (Bglap)	0.57	0.57	0.00	9.33E-01
Dentin matrix acidic phosphoprotein 1 (Dmp1)	0.37	0.51	0.46	4.05E-01
Alkaline phosphatase, tissue-nonspecific isozyme (Alpl)	0.15	0.39	1.38	7.43E-02
Osteocalcin-related protein (Bglap3)	0.12	0.18	0.58	7.19E-01
Osteocrin (Ostn)	0.01	0.09	3.17	3.28E-01
Osteomodulin (Omd)	0.04	0.06	0.58	7.19E-01
Runt-related transcription factor 2 (Runx2)	11.16	6.42	−0.80	1.51E-20

### The accuracy of RNA-seq results was further consolidated by qRT-PCR assay

**Fig. 5:**
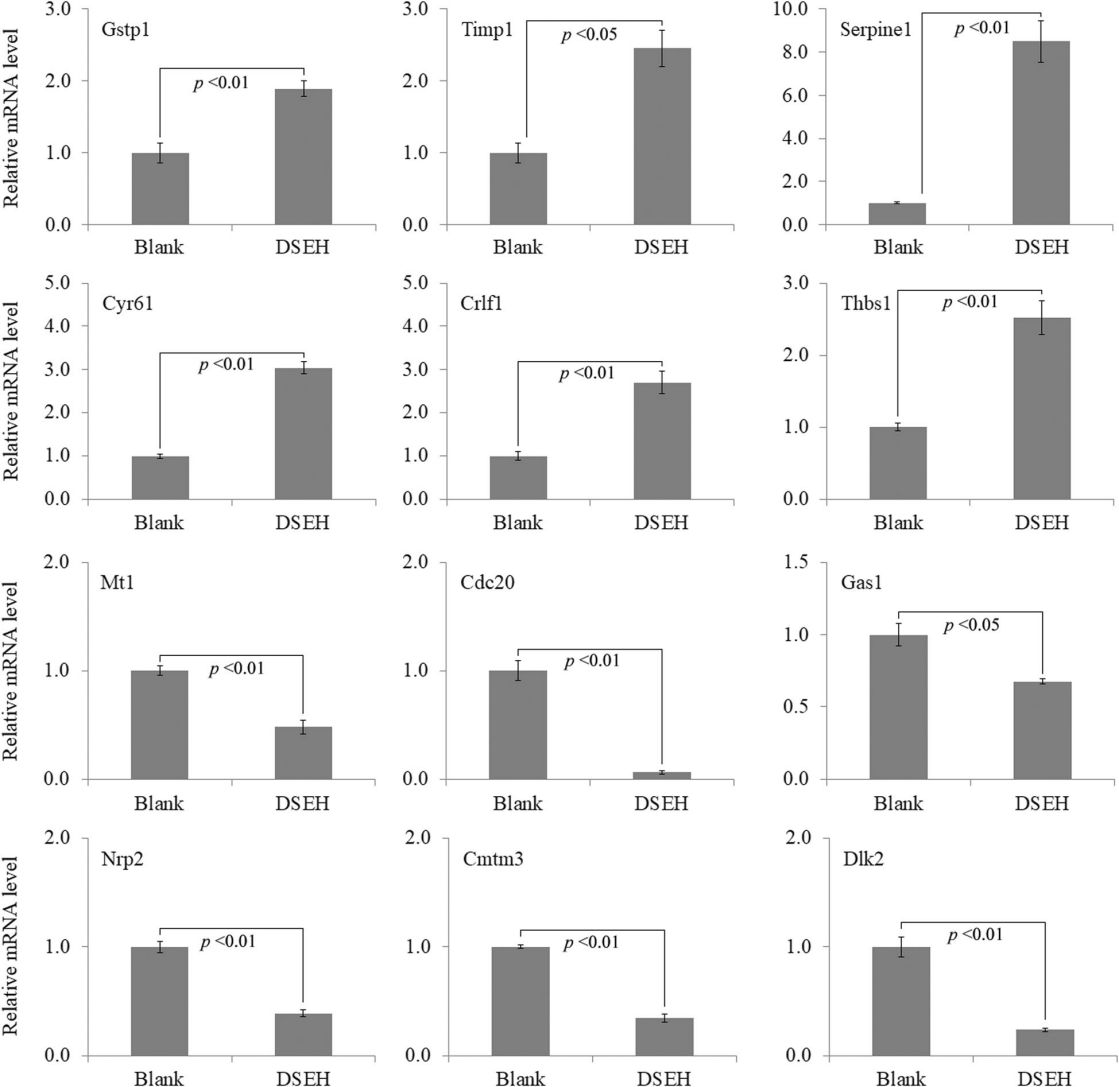
Verification of RNA-seq data by qRT-PCR assay. The relative mRNA levels of the selected DEGs were quantified by qRT-PCR. Data were presented as the mean with standard deviation for technical triplicate in an experiment representative of several independent ones. *p* < 0.05 and *p* < 0.01 represented the differences of relative mRNA levels under DSEH treatment in a student t-test

To further verify and consolidate the accuracy of the RNA-seq data, 12 DEGs including 6 up-regulated genes (Gstp1, Timp1, Serpine1, Cyr61, Crlf1 and Thbs1) and 6 down-regulated genes (Mt1, Cdc20, Gas1, Nrp2, Cmtm3 and Dlk2) were chosen to verify the accuracy of RNA-seq results by analyzing their expression levels using qRT-PCR assay. Gene specific primer sequences were shown in Table 8. The relative mRNA expression levels were presented in Fig. 5. The results showed that the qRT-PCR assay obtained consistent results with those obtained by RNA-seq analysis.

**Table 8:** List of qRT-PCR primers for DEGs

Gene name	Primer	Sequence
Gstp1	Forward primer	ATGCCACCATACACCATTGTC
	Reverse primer	GGGAGCTGCCCATACAGAC
Timp1	Forward primer	GCAACTCGGACCTGGTCATAA
	Reverse primer	CGGCCCGTGATGAGAAACT
Serpine1	Forward primer	TTCAGCCCTTGCTTGCCTC
	Reverse primer	ACACTTTTACTCCGAAGTCGGT
Cyr61	Forward primer	CTGCGCTAAACAACTCAACGA
	Reverse primer	GCAGATCCCTTTCAGAGCGG
Crlf1	Forward primer	CTCCCTGCAAGCTACCTGC
	Reverse primer	AGGGTGGAGGTGTTAAGGAGG
Thbs1	Forward primer	GGGGAGATAACGGTGTGTTTG
	Reverse primer	CGGGGATCAGGTTGGCATT
Mt1	Forward primer	GAGTCTGTAGTTGTCGCAGTTG
	Reverse primer	GGCCTTTTTGGAAGGAAGAGGA
Cdc20	Forward primer	TTCGTGTTCGAGAGCGATTTG
	Reverse primer	ACCTTGGAACTAGATTTGCCAG
Gas1	Forward primer	CCATCTGCGAATCGGTCAAAG
	Reverse primer	GCTCGTCGTCATATTCTTCGTC
Nrp2	Forward primer	GCTGGCTACATCACTTCCCC
	Reverse primer	CAATCCACTCACAGTTCTGGTG
Cmtm3	Forward primer	GCCGAGTCGGGTCTTTCATTC
	Reverse primer	GAGGAAGTAAACGGCCAACAG
Dlk2	Forward primer	GGCCAGTGTGTGTATGACGG
	Reverse primer	CGGCATGTGAAGTTGAGGG
Gapdh	Forward primer	GCACAGTCAAGGCCGAGAAT
	Reverse primer	GCCTTCTCCATGGTGGTGAA

### DSEH inhibited MC3T3-E1 cell apoptosis caused by H_2_O_2_-induced oxidative injury

**Fig. 6:**
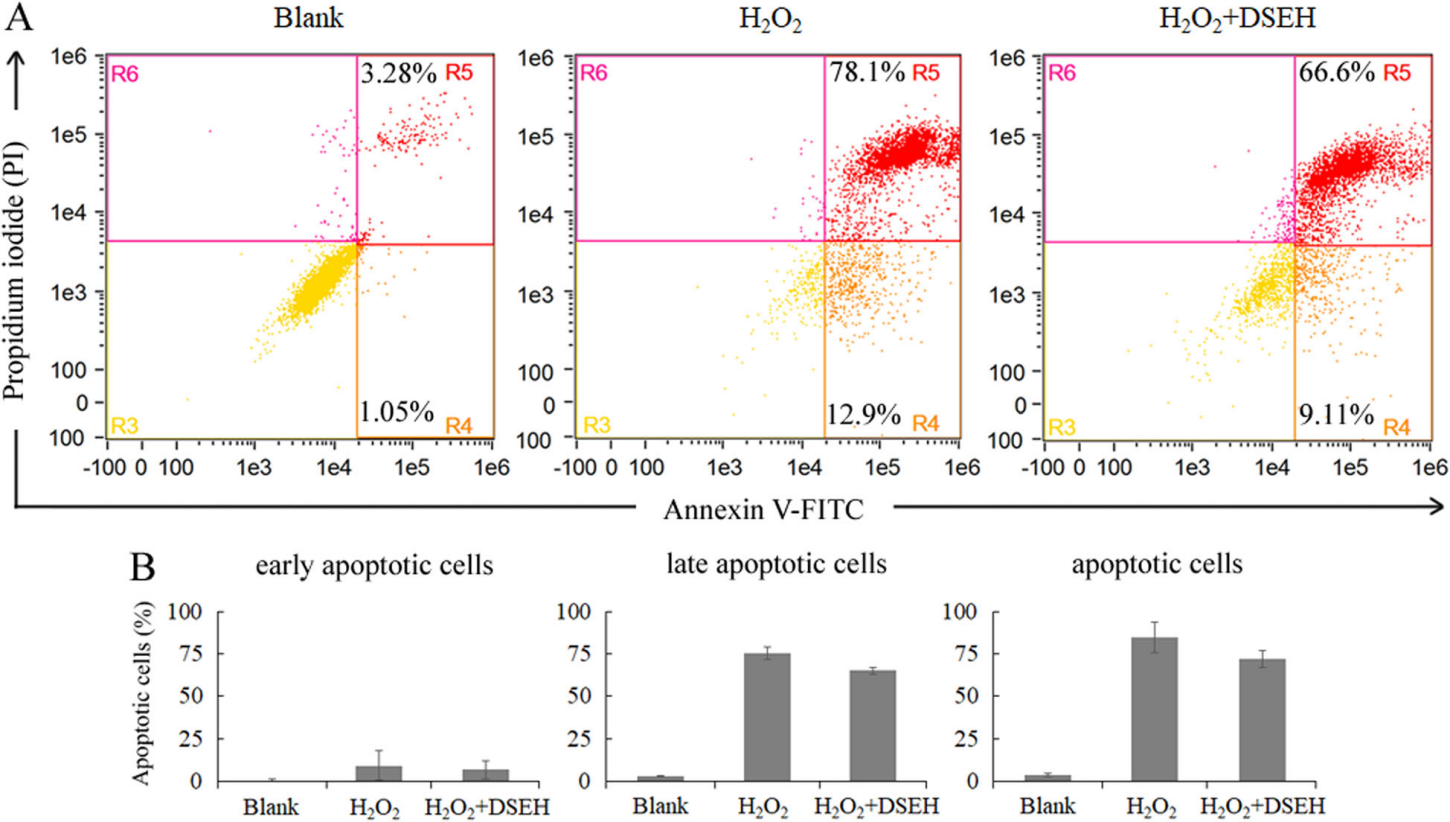
Effects of DSEH on cell apoptosis caused by H_2_O_2_-induced oxidative injury. Cell apoptosis was assessed by staining the cells with Annexin V and PI and performing on a flow cytometry instrument. In each graph, the lower left region (R3) represents viable cells (Annexin V (−) PI (−)); the lower right region (R4) represents early apoptotic cells (Annexin V (+) PI (−)); the upper right region (R5) represents late apoptotic cells (Annexin V (+) PI (+)); and the upper left region (R6) represents necrotic cells (Annexin V (−) PI (+)). H_2_O_2_: hydrogen peroxide

In order to confirm the results obtained by cell proliferation assay, Alizarin red staining and RNA-seq analysis, effects of DSEH on MC3T3-E1 cells under H_2_O_2_-induced oxidative injury were analyzed using Annexin V/PI staining and flow cytometry following 24 h of incubation with DSEH. As shown in Fig. 6, compared with the negative control group (Blank), the percentage of apoptotic cells (R4: early apoptotic cells; R5: late apoptotic cells) were increased under H_2_O_2_ induction (H_2_O_2_). However, DSEH treatment (H_2_O_2_ + DSEH) decreased the percentage of apoptotic cells, especially the late apoptotic cells.

## Discussion

Sinew shares a series of types of collagen components with bone, including type I, III and V collagens, of which type I collagen is expressed in the whole process of bone growth and development, and type III collagen play pivotal role in regulating bone regeneration during the process of bone repair [25]. Deer Sinew serves as a medicinal food, and has been used for treating skeletal diseases, especially bone diseases in a long history. Thus, it could become an alternative option for the prevention and therapeutic remedy of bone-related diseases. However, the exact regulation mechanism remains to be elucidated. In the present study, the results of CCK-8 assay showed that DSEH promoted the proliferation of both MC3T3-E1 cells and primary osteoblasts in a dose-dependent manner. Based on the analysis of Alizarin red staining and quantification, DSEH significantly enhanced extracellular matrix synthesis of MC3T3-E1 cells at day 1 and day 3 with a similar pattern, but inhibit extracellular matrix synthesis of MC3T3-E1 cells from day 7 to day 13. However, DSEH showed no significant effects on extracellular matrix synthesis of primary osteoblasts. Therefore, we further explored the molecular regulation of DSEH on the gene expression of MC3T3-E1 cells using RNA-seq technique together with qRT-PCR verification method, and demonstrated that DSEH promoted the proliferation of MC3T3-E1 cells and suppressed the apoptosis of those cells by regulating multiple functional genes.

According to our findings, we addressed that DSEH promoted osteoblast proliferation and extracellular matrix synthesis by increasing the expression levels of genes positively regulating the proliferation of MC3T3-E1 cells, including Gstp1, Timp1, Serpine1, Cyr61, Crlf1, Thbs1, Ctgf, P4ha2, Sod3 and Nqo1. Among these genes, Gstp1 is a glutathione transferase that acts as a regulator of signaling pathways that are involved in cell proliferation, and contributes in protection against osteoblast apoptosis [26]. Timp1, a member of the tissue inhibitor of metalloproteinases family, increases osteoblast proliferation and decreased apoptosis [27]. Serpine1 is a primary inhibitor of endogenous plasminogen activators, which serves as a regulator during cell growth and cell proliferation, and plays an important role in regulating resorption during bone fracture repair [28]. Cyr61 is a cysteine-rich angiogenic inducer that is capable of regulating a broad range of cellular activities, such as cell adhesion and proliferation, and could stimulate osteoblast proliferation during bone remodeling [29]. Crlf1 is a member of the ciliary neurotrophic factor receptor pathway, and is involved in the anabolic therapy of osteoporosis through regulating osteoblast proliferation and bone formation [30]. Thbs1 is an adhesive glycoprotein that is directly deposited by bone-forming osteoblasts, and is present in the mineralized bone matrix [31]. Ctgf is an extracellular matrix-associated growth factor that plays a pivotal role in osteoblast proliferation and bone formation [32]. P4ha2 is a subunit of collagen prolyl-4-hydroxylase that is mainly expressed in osteoblasts, chondrocytes and capillary endothelial cells, and serves as an important enzyme during collagen biosynthesis [33]. Sod3 is a member of the superoxide dismutase protein family, and has a protective effect on oxidative stress-induced damage in osteoblastic cells [34]. Nqo1 is a pleiotropic enzyme that is expressed in calvarial osteoblasts, and protects the cells from oxidative damage [35]. Thus, these findings suggest that DSEH facilitate osteoblast proliferation, maintain osteoblast homeostasis and protect oxidative stress-induced damage.

We next addressed that DSEH promoted osteoblast proliferation and extracellular matrix synthesis by decreasing the expression levels of genes negatively regulating the proliferation of MC3T3-E1 cells, including Mt1, Cdc20, Gas1, Nrp2, Cmtm3, Dlk2, Sema3a, Rbm25 and Hspb6. Among these genes, Mt1 is a family member of the cysteine-rich metallothioneins, and is mainly expressed during osteoblastic differentiation and mineralization [36]. Cdc20 is an activator of the anaphase-promoting complex, and is weakly expressed in osteoblasts but highly expressed in the osteocytes [37]. Gas1 is a cell growth repressor gene that serves as a suppressive regulator of cell growth and as a mediator of apoptosis [38]. Nrp2 is a family member of the neuropilins, and one of its isoform Nrp2b inhibits cultured cell proliferation [39]. Cmtm3, a member of the chemokine-like factor gene superfamily, is an inhibitor of cell growth, and an inducer of cell apoptosis [40]. Dlk2 is a member of the EGF-like family of membrane proteins, and interacts with Notch signaling to regulate cell growth and apoptosis [41]. Sema3a is a member of the semaphoring family, which suppresses cell proliferation and promotes cell apoptosis [42]. Rbm25, a RNA-binding protein, negatively regulates cell proliferation and positively regulating cell apoptosis [43]. Hspb6, also known as Hsp20, is a small heat shock protein that participates in reducing cell proliferation and inducing cell apoptosis [44]. Thus, these findings suggest that DSEH prevent osteoblast apoptosis, maintain osteoblast homeostasis and prevent osteoblasts form further differentiation.

We further addressed that DSEH mildly increased the expression levels of a majority of the osteoblast markers, including Spp1, Col1a1, Fn1, Atf4, Bst2, Bmp1, Bmp4, Sox4, Sox11, Msx1, Znf25, Bglap2, Sp7, Bglap, Dmp1, Alpl, Bglap3, Ostn and Omd. Among these genes, Spp1, Col1a1, Fn1, Bglap, Bglap2, Bglap3 and Dmp1 are the major bone matrix protein genes [45–47]. Atf4, Sox4, Sox11, Msx1, Znf25 and Sp7 are key transcription factors involved in osteoblast commitment, proliferation, differentiation and extracellular matrix synthesis during bone formation [48–52]. Bst2, also known as tetherin, is a lipid raft associated protein that is highly expressed in osteoblasts, and is involved in the process of osteogenic differentiation [53]. Bmp1 and Bmp4, two members of the bone morphogenetic protein family, are important stimulator of osteoblast proliferation and differentiation, bone formation and stability [54, 55]. Alpl is a well-known osteoblastic marker, and has been used as an evaluation indicator to estimate the capacity of bone formation [56]. Ostn is a secreted bone-active protein that is highly expressed in osteoblasts, and plays key role in regulating osteoblast activity in developing bone and at the sites of bone remodeling [57]. Omd, also known as osteoadherin, is mainly expressed in osteoblasts at early differentiated stage, and serves as an organizer of bone mineral formation [58]. However, the expression level of Runx2 was mildly decreased under DSEH treatment, and the underlying molecular mechanism still need to be further studied. Thus, these findings suggest that DSEH facilitate osteoblast proliferation, differentiation and extracellular matrix synthesis to consolidate bone formation and stability.

Consistent with the above results, our enrichment analysis of GO function and KEGG pathway indicated that the DEGs were primarily classified into the cellular components of extracellular matrix, intracellular part and organelle with dominant molecular functions of binding activities, and predominantly participated in the biological processes including regulation of metabolic process, single-multicellular organism process and developmental process. Those DEGs were mainly mapped to the signaling pathways such as thyroid hormone signaling pathway, regulation of actin cytoskeleton, PI3K-Akt signaling pathway, FoxO signaling pathway, focal adhesion, ECM-receptor interaction, cAMP signaling pathway and axon guidance. Among these pathways, thyroid hormone signaling pathway plays a key role in the regulation of bone metabolism by modulating osteoblast activity during skeletal development [59]. Actin cytoskeleton has been considered to be involved in various functions of osteoblast by discerning cell condition and regulating extracellular matrix mineralization and bone formation [60]. PI3K-Akt signaling pathway is a central regulation hub for osteoblast homeostasis and function, and has the capacity to work together with other signaling networks to regulate osteoblast phenotype and bone formation [61]. FoxO signaling is indispensable for bone mass homeostasis since this pathway could prevent oxidative stress and osteoblast apoptosis, and promote osteoblast proliferation to facilitate bone formation and stability [62]. Focal adhesion facilitates the perpendicular arrangement of bone matrix orthogonal to osteoblast alignment during bone formation [63]. ECM-receptor interaction plays a crucial role in regulating osteoblast adhesion, proliferation and survival during bone formation and repair [64]. cAMP signaling pathway is capable of enhancing cell adhesion of osteoblasts to facilitate bone regeneration [65]. Axon guidance signaling pathway plays a pivotal role in regulating bone formation and resorption by promoting osteoblast migration and suppressing osteoclast differentiation [66]. Finally, the results of DSEH on osteoporotic model caused by hydrogen peroxide (H2O2)-induced oxidative injury further indicated that DSEH treatment decreased the percentage of apoptotic cells, especially the late apoptotic cells. Thus, these findings confirmed that DSEH facilitated osteoblast proliferation and extracellular matrix synthesis, and prevented osteoblast oxidative stress and apoptosis to consolidate bone formation and repair.

## Conclusions

In summary, we performed cell proliferation assay, Alizarin red staining and quantification, RNA-seq analysis accompanied with verification methods to dissect the molecular mechanism of DSEH in the regulation of an osteoblast-like cell line MC3T3-E1. Our findings suggest that DSEH facilitate osteoblast proliferation and extracellular matrix synthesis to consolidate bone formation and stability, but prevent osteoblasts from oxidative stress-induced damage, apoptosis and further differentiation. These findings deepened the current understanding of DSEH on regulating bone development, and provided theoretical support for the discovery of optional prevention and treatment for bone-related diseases.

## Data Availability

The datasets used and/or analyzed during the current study are available from the corresponding author on reasonable request.

## Abbreviations

DSEH: Deer sinews enzymatic hydrolysate
RNA-seq: RNA sequencing
RIN: RNA integrity number
BLAST: Basic Local Alignment Search Tool
DEG: Differentially expressed genes
qRT-PCR: Quantitative real-time PCR
SRA: Sequence Read Archive
GO: Gene ontology
Gapdh: Glyceraldehyde 3-phosphate dehydrogenase
KEGG: Kyoto Encyclopedia of Genes and Genomes
